# Validation of bidimensional measurement in nasopharyngeal carcinoma

**DOI:** 10.1186/1748-717X-5-72

**Published:** 2010-08-16

**Authors:** Ting-Shou Chang, Sau-Tung Chu, Yu-Yi Hou, Kuo-Ping Chang, Chao-Chuan Chi, Ching-Chih Lee

**Affiliations:** 1 Department of Otolaryngology, Kaohsiung Veterans General Hospital, Kaohsiung, Taiwan; 2 Department of Otolaryngology, Buddhist Tzu Chi Dalin General Hospital, Chiayi County 622, Taiwan; 3 School of Medicine, Tzu Chi University, Hualian, Taiwan

## Abstract

**Background:**

Our previous study showed a close relationship between computed tomography (CT)-derived bidimensional measurement of primary tumor and retropharyngeal nodes (BDMprn) and gross tumor volume of primary tumor and retropharyngeal nodes (GTVprn) in nasopharyngeal carcinoma (NPC) and better prognosis for NPC patients with smaller BDMprn. In this study, we report the results on of a study to validate the use of BDM in a separate cohort of NPC patients.

**Methods:**

We retrospectively reviewed 103 newly diagnosed NPC cases who were treated with radiotherapy/concurrent chemoradiotherapy (CCRT) or CCRT with adjuvant chemotherapy from 2002 to 2009. We used magnetic resonance imaging (MRI) to measure BDMprn. We calculated overall survival, recurrence-free and distant metastasis-free survival curves and set a BDMprn cut off point to categorize patients into a high- or low-risk group. We then used Cox proportional hazard model to evaluate the prognostic influence of BDMprn after correcting age, gender and chemotherapy status.

**Results:**

After adjusting for age, gender, and chemotherapy status, BDMprn remained an independent prognostic factor for distant metastasis [Hazard ratio (HR) = 1.046; *P *= 0.042] and overall survival (HR = 1.012; *P *= 0.012). Patients with BDMprn < 15 cm^2 ^had a greater 3-year overall survival rate than those with BDMprn ≧ 15 cm^2 ^(92.3% vs. 73.7%; *P *= 0.009). They also had a greater 3-year distant metastasis-free survival (94% vs.75%; *P *= 0.034).

**Conclusion:**

The predictive ability of BDMprn was validated in a separate NPC cohort. A BDMprn of 15 cm^2 ^can be used to separate NPC patients into high- and low-risk groups and predict survival rates and metastasis potential. It can, therefore, be used as a reference to design clinical trials, predict prognosis, and make treatment decisions.

## Background

Nasopharyngeal carcinoma (NPC) is common among Asians, especially in southern China. While the annual incidence in Western countries is < 1 per 100,000 population, it is 6.17 per 100,000 in Taiwan [1]. Because it is difficult to approach nasopharyngeal tumors surgically, chemoradiotherapy or radiotherapy is the primary means of treating this disease [2]. The American Joint Committee of Cancer (AJCC) staging system for NPC is widely used to prognosticate and plan for its treatment and is well-accepted as an evaluation tool in clinical research. However, because the current TNM staging approach is limited in its ability to predict prognosis based on NPC tumor stage [3,4], other factors might be incorporated to further refine prognostic accuracy.

Gross tumor volume is one factor closely related to NPC survival [5-8]. It is not, however, widely advocated as a prognostic factor probably because measuring tumor volume can be time-consuming and labor-intensive. Several studies have used unidimensional and bidimensional measurement to evaluate the tumor size [9-11]. In a previous study, we found bidimensional measurement of primary NPC tumor and retropharyngeal nodes by computed tomography (CT) imaging to be an independent prognostic factor [12]. Due to its improved accuracy, magnetic resonance imaging (MRI) has now virtually replaced CT scan as means of determining the stage of tumors, including NPC, before they are treated [13]. MRI is superior to CT scan for diagnosing the gross extent of tumor infiltration and retropharyngeal lymph node metastasis.

In this study, we retrospectively reviewed MRI images in a separate cohort of NPC patients to further validate of the previous finding regarding the use of bidimensional measurement as means of prognosis in NPC. If confirmed to be an independent prognostic factor, then prognostic ability of the current TNM staging approach can be improved.

## Methods

### Patient selection

The method of bidimensional measurement of primary tumor and retropharyngeal nodes (BDMprn) in NPC was derived from a cohort of newly diagnosed NPC patients with definite treatment [12]. All patients had histological confirmed NPC and received CT scan of the nasopharyngeal area, chest X-ray, ultrasound or CT scan of the abdomen and whole body bone scan. All cases were restaged based on criteria outlined in the 6th edition of the AJCC staging system [14] Patients received a complete course of radiotherapy (70 Gy - 75 Gy). Patients who received concurrent chemoradiotherapy (CCRT) received three cycles of cisplatin during the same period that were undergoing radiotherapy. Subsequent adjuvant chemotherapy consisting of cisplatin and 5-FU was arranged as guidelines [2]. Using computed tomography-derived measurement, bidimensional measurement of primary tumor and retropharyngeal nodes (BDMprn) in NPC had good correlation with gross tumor volume (Spearman' correlation coefficient = 0.845, P < 0.001). The intrarater reliability for BDM was good. In multivariate analysis, BDMprn was an independent prognostic factor for any relapse [Hazard ratio (HR) = 1.066; P = 0.029], and overall survival [HR = 1.097; P = 0.007]. NPC patients with large BDMprn conferred a poor survival and more recurrences[12].

Validation of the bidimensional measurement of primary tumor and retropharyngeal nodes was performed using a cohort which included NPC patients treated at Kaohsiung Veterans General Hospital from 2002 to 2009. The means of treating NPC patients in these two hospitals is similar. All patients received a complete course of radiotherapy (70 Gy - 75 Gy). Concurrent chemotherapy was arranged for NPC patients with advanced T (T2-4) classification or positive neck metastasis. Patients with T2b-T4 or N2-3 disease underwent subsequent adjuvant chemotherapy.

Before treatment, all NPC patients received physical examinations, fiberoptic examinations, chest X-rays, ultrasound or CT scan of the abdomen, whole body bone scan and MRI of nasopharyngeal area. Similarly, all cases were restaged according to the AJCC stage classification system, which was modified in 2002.

### MRI technique and measurements

**Figure 1:**
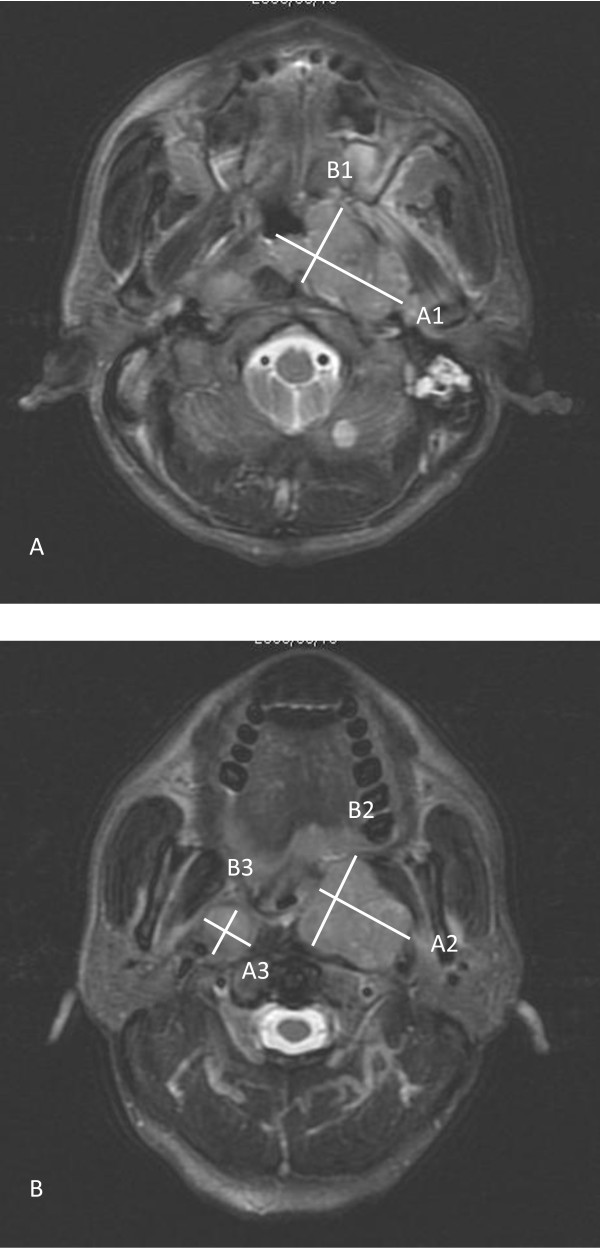
**T2-weighted postcontrast MR image in the axial plane**. The bidimensional measurement of primary tumor and retropharyngeal nodes (BDMprn) was obtained by summation of multiplying the maximum diameter by the greatest measurement perpendicular to it in nasopharyngeal tumor (A) and retropharyngeal nodes (B). BDMprn (cm^2^) = A1 × B1 + A2 × B2 + A3 × B3.

Gross tumor volume of primary tumor and retropharyngeal nodes (GTVprn) of NPC measurement was performed with summation of area technique as described previously [12]. The lateral retropharyngeal nodes were considered malignant if its shortest axial dimension was 5 mm or greater, and any visible node in the median retropharyngeal group was considered metastatic [15-17]. Bidimensional measurement of primary tumor and retropharyngeal nodes (BDMprn) was performed as described previously [12]. Briefly, BDMprn was obtained by multiplying the maximum diameter of the nasopharyngeal tumor and retropharyngeal nodes by the greatest measurement perpendicular to it (Figure 1). Bidimensional measurement of primary tumor (BDMp) was calculated by multiplying the maximum diameter of the nasopharyngeal tumor by the greatest measurement perpendicular to it [18]. It was sometimes difficult to evaluate the anatomic extent of primary tumor and retropharyngeal node. In such cases, when the outline of tumor was unclear, a radiologist specializing in head and neck cancer helped demarcate the margin. When there was skull base involvement or parapharyngeal space invasion, we could measure the gross tumor and retropharyngeal nodes using the same methods in Figure 1.

The calculation of the three measurements was as the followings:

GTVprn = Ʃ Outlined area of primary tumor and retropharyngeal nodes × (slice thickness + split interval).

BDMprn = Ʃ Maximum diameter × greatest perpendicular of primary tumor and retropharyngeal nodes.

BDMp = Maximum diameter × greatest perpendicular of primary tumor.

### Clinical endpoints

Clinical endpoints were 3-year overall survival, any recurrence and distant metastasis. Six weeks after completing the course of treatment, patients received endoscopy and biopsy of the nasopharynx if necessary. Two months after the course of treatment, each patient received a MRI examination. Chest X-rays, abdominal sonography, and whole body bone scan were performed regularly.

### Statistics

Intrarater reliability was measured using the intraclass correlation coefficient. Overall survival, distant metastasis-free survival and recurrence-free survival were calculated according to the methods of Kaplan and Meier. Differences between multiple survival curves were compared using the log-rank test. The prognostic influence of BDM was assessed using Cox proportional hazards multivariate model after adjusting for age, gender, and chemotherapy status. BDM cut-off values were obtained by receiver operating characteristic (ROC) curve analysis. All statistical operations were performed using the Statistical Package for Social Sciences, version 15.0 (SPSS, Chicago, IL).

## Results

### Patient and disease characteristics

The intrarater reliability correlation coefficients for GTVprn, BDMprn, and BDMp were 0.956(0.935-0.97), 0.964 (0.912-0.986), and 0.966 (0.916-0.987). Table 1 shows the characteristics of patients in validation cohort. The mean age was 51 ± 13 years. Of the 103 NPC patients, 77 (75%) patients were men. 88 patients (85%) had advanced stage (stage III-IV). These NPC patients were followed up a median of 43 months (range 9-80 months). Thirty-four (33%) in the validation cohort had recurrences, including 15 (15%) with locoregional recurrence and 12 (12%) with distant metastasis. Eighteen patients (18%) expired. The 3-year overall survival rate was 87%, locoregional control survival rate 88%, distant metastasis-free survival rate 89%, and recurrence-free survival rate 79%.

**Table 1:** Patient Characteristics

Variables	Validation cohort (*n *= 103) No. of patients (%)
Age (years)	
Mean ± SD	51 ± 13
Gender	
Male	77(75)
Female	26(25)
Stage	
I	3(3)
II	12(12)
III	56(54)
IV	32(31)
T classification	
T1	25(24)
T2	20(19)
T3	34(33)
T4	24(23)
N classification	
N0	6(6)
N1	11(11)
N2	72(70)
N3	14(14)
Histology grade	
Non-keratinizing carcinoma	13(13)
Undifferentiated carcinoma	90(90)
Treatment modality	
RT/CCRT	76(74)
CCRT+CT	27(26)

SD, standard deviation; RT, radiotherapy; CCRT, concurrent chemoradiotherapy; CCRT+CT, concurrent chemoradiotherapy with adjuvant chemotherapy

### Univariate and multivariate analysis

Based on our univariate analysis, bidimensional measurement of primary tumor and retropharyngeal nodes was found to be a significant prognostic factor (Table 2). Adjusting for age, gender, and chemotherapy status, our multivariate analysis found bidimensional measurement of primary tumor and retropharyngeal nodes to significantly predict overall survival (HR = 1.012; 95% CI: 1.014-1.12; P = 0.012) and metastasis-free survival (HR = 1.046; 95% CI: 1.002-1.121; P = 0.042). The bidimensional measurement of primary tumor was not a significant predictor for outcomes in multivariate analysis. Both univariate and multivariate analysis found gross tumor volume of primary tumor and retropharyngeal nodes to be a significant prognostic factor.

**Table 2:** Univariate and multivariate analysis results (*n *= 103)

	Overall survival		Metastasis		Any recurrence	
	
	Univariate HR (95% CI)	Multivariate* HR (95% CI)	Univariate HR (95% CI)	Multivariate* HR (95% CI)	Univariate HR (95% CI)	Multivariate* HR (95% CI)
GTVprn	1.07 (1.037-1.103)	1.069 (1.033-1.107)	1.049 (1.013-1.085)	1.05 (1.01-1.091)	1.048 (1.018-1.079)	1.037 (1.004-1.071)
	P < 0.001	P < 0.001	P = 0.007	P = 0.013	P = 0.002	P = 0.028
BDMprn	1.071 (1.021-1.122)	1.012 (1.014-1.12)	1.06(1.006-1.116)	1.06 (1.002-1.121)	1.046 (1.002-1.093)	1.038 (0.99-1.088)
	P = 0.004	P = 0.012	P = 0.028	P = 0.042	P = 0.041	P = 0.121
BDMp	1.056 (1.001-1.113)	1.048 (0.991-1.108)	1.041 (0.983-1.116)	1.043 (0.981-1.108)	1.043 (0.993-1.096)	1.037 (0.984-1.092)
	P = 0.045	P = 0.099	P = 0.169	P = 0.176	P = 0.091	P = 0.173

GTVprn, gross tumor volume of primary tumor and retropharyngeal nodes; BDMprn, bidimensional measurement of primary tumor and retropharyngeal nodes; BDMp, bidimensional measurement of primary tumor; HR, hazard ratio; 95% CI, 95% confidence interval.

*Multivariate analysis: adjusted for age, gender, and chemotherapy status.

### Bidimensional measurement and risk groups

**Figure 2:**
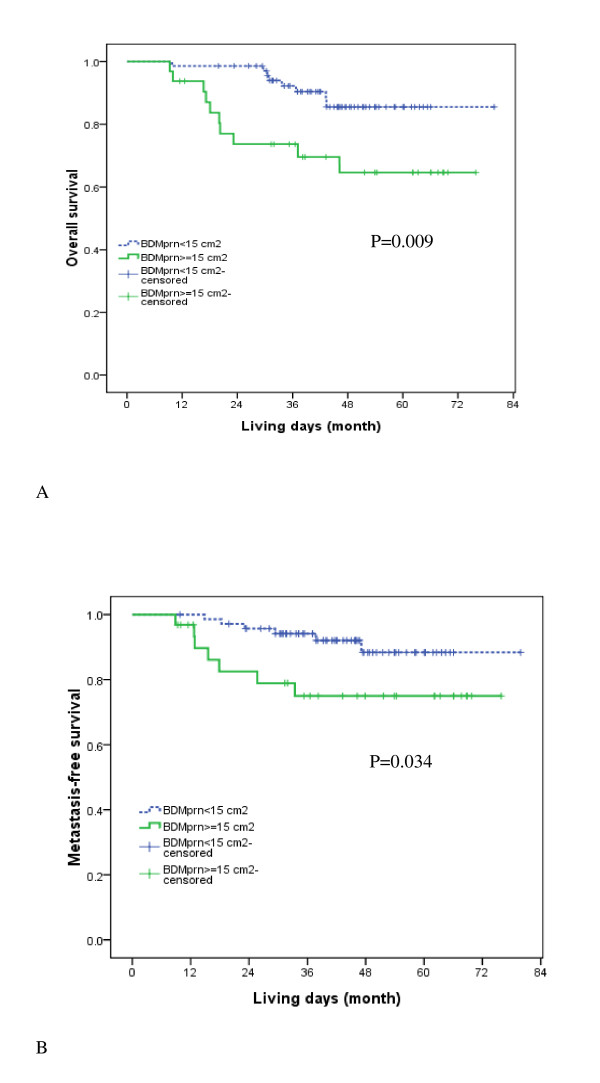
**Survival curves**. (A)Probability of overall survival rates by small versus large BDMprn. (B) Probability of distant metastasis-free survival rates by BDMprn.

We wanted to further validate the prognostic ability of bidimensional measurement of primary tumor and retropharyngeal nodes using MRI findings. After analyzing trade-off, we chose 15 cm^2 ^as the cut-off point in the validation cohort (additional file 1). Using this cut-off point, we further divided validation cohort into a smaller BDMprn group (67%) and a larger BDMprn group (33%). The smaller BDMprn group had greater 3-year overall survival, distant metastasis-free survival, and recurrence-free survival rates than the large BDM group. (92.3% vs. 73.7%, P = 0.009; 94% vs. 75%, P = 0.034; 64.1% vs. 59.7%, P = 0.082) (Figure. 2A and 2B), and they were at lower risk.

## Discussion

In a previous study, CT-derived bidimensional measurement of primary tumor and retropharyngeal nodes could be used to predict prognosis of NPC [12]. Using MRI to validate the ability of bidimensional measurement of primary tumor and retropharyngeal nodes to predict NPC outcomes in a validation cohort, we found BDMprn remained an independent prognostic factor for overall survival as well as metastasis-free survival. Adopting a BDMprn of 15 cm^2 ^as cut-off point in validation cohort, NPC patients whose BDMprn was less than 15 cm^2 ^had a better 3-year overall survival rate and distant metastasis-free survival rate than those with BDMprn above this cut off point. Based on these two studies, we have found that BDMprn can be used to stratify patients into two different prognostic groups with significantly different overall survival and metastatic rates.

Although the current TNM staging system for NPC is widely used, it has been reported to have several deficiencies. Mao *et al. *[3] and Cheng *et al. *[4] have not found any significant differences in local-relapse free survival among the T1, T2, and T3 NPC subgroups. Recently, gross tumor volume has been reported to be a risk factor for local recurrence of NPC [5,6,19]. However, measurement of gross tumor volume is time-consuming, and the technology, expertise, and manpower are often not available in routine clinical practice.

In a study of bidimensional and unidimensional MRI-derived measurement to reflect NPC tumor anatomic extent at diagnosis or the change in size after treatment, King *et al. *[18] found that BDM of primary tumor was a quicker and more widely applicable method than tumor volume measurement and that it could be used to assess tumor response. However, measurement of retropharyngeal nodes were not included in that series. Tang *et al. *[20] showed that retropharyngeal lymph node metastasis affects the distant metastasis-free survival rates of NPC, and Wang *et al. *[21] found a good correlation between retropharyngeal lymph node metastasis and parapharyngeal space involvement as well as metastasis to Level II, III, IV and/or V nodes. Based on these findings, our previous study modified the approach used by King et al. to include both primary tumor and retropharyngeal lymph nodes measurements in our definition of BDM. Previous study found a very close relationship between CT-derived BDMprn, gross tumor volume of primary tumor and retropharyngeal nodes, and overall survival [12]. In the present study, also incorporating retropharyngeal lymph node measurements, we found MRI-derived BDMprn could also predict overall survival as well as metastasis free survival in NPC patients.

In our study, we found that we could use BDMprn to categorize patients into low- and high-risk groups. This distinction would facilitate treatment decisions, as it would spare low-risk NPC patients from receiving aggressive treatment. Although NPC is markedly radiosensitive, there is a high failure rate in treatment due to its metastatic behavior. Improvement in the outcome for NPC relies on the delivery of higher radiation doses [22]. While radiotherapy is the only standard treatment for early-stage NPC (stage I), the combination of cisplatin-based chemotherapy and radiotherapy is used to treat patients with advanced NPC (stage II-IVB) [2]. The latter group not only receives higher doses of radiotherapy, they also receive chemotherapy, both associated with significant comorbidity, including myocardial infarction, severe nutrient deficiency, nephrotoxicity, transverse myelitis, leukopenia, and central nervous system disease [2,23,24]. Recent study revealed that NPC patients with GTVprn ≧ 13 ml conferred a poor prognosis and may benefit from ≧ 4 cycles of chemotherapy [25]. This series implied that high-risk NPC patients, such as large GTVprn, could benefit from more intensive chemotherapy and radiotherapy. The treatment goals for NPC is to adjust chemoradiotherapy dosages to achieve adequate anticancer effects without overly increasing the development of such complications. It would be important and valuable if high-risk NPC patients could be identified in order to adjust the radiation dose and tailor chemotherapy protocol. In this way, high-risk patients (larger BDMprn) may benefit from more extensive treatment approaches, such as more intensive chemotherapy or higher dose of radiation, whereas low-risk patients (smaller BDMprn) may do well with standard therapy and can be spared the severe toxic side effects of more radical therapy.

## Conclusion

We have validated bidimensional measurement of primary tumor and retropharyngeal nodes in a different cohort of NPC with pretreatment staging by MRI. BDMprn, derived by MRI, is closely related to survival rates and metastatic rates of NPC patients. BDMprn can stratify patients into two different prognostic groups with significantly different overall survival. Nasopharyngeal carcinoma patients with large bidimensional measurement have poor survival rates and high metastasis potential. BDMprn might be used in the future for the design of clinical trials, the prediction of survival, and treatment decisions.

## Competing interests

The authors declare that they have no competing interests.

## Authors' contributions

TSC and CCL designed the study, collected the data, interpreted the results of the study, and oversaw the project completion. STC, YYH, KPC and CCC participated in preparing of data acquisition. TSC and CCL performed the statistical analysis and drafted the manuscript. All authors contributed to the scientific setup of the study and revised the manuscript critically, and they have approved the final version of the manuscript.

## Supplementary Material

Additional file 1 **Table S1**. Validity of BDMprn using death, distant metastasis or any recurrence as the standard. Click here for file
