# Supplementary material for: Validation of bidimensional measurement in nasopharyngeal carcinoma
Source: Radiat Oncol. 2010 Aug 16;5:72. doi: 10.1186/1748-717X-5-72 (PMC2930639; doi:10.1186/1748-717X-5-72)
Supplement: Additional file 1 — Table S1. Validity of BDMprn using death, distant metastasis or any recurrence as the standard. [file 1748-717X-5-72-S1.DOCX]

| Table S1. Validity of BDMprn using death, distant metastasis or any recurrence as the standard | | | | | | | | |  |
| --- | --- | --- | --- | --- | --- | --- | --- | --- | --- |
|  | Overall survival | |  | Distant metastasis | |  | Any recurrence | | |
| Cut-off point | Sensitivity | Specificity |  | Sensitivity | Specificity |  | Sensitivity | Specificity | |
| 10 cm^2^ | 0.667 | 0.553 |  | 0.615 | 0.533 |  | 0.56 | 0.538 | |
| 13 cm^2^ | 0.556 | 0.694 |  | 0.538 | 0.678 |  | 0.44 | 0.679 | |
| **15 cm^2^** | **0.556** | **0.741** |  | **0.538** | **0.722** |  | **0.44** | **0.731** | |
| 20 cm^2^ | 0.222 | 0.906 |  | 0.308 | 0.961 |  | 0.2 | 0.91 | |
|  | | | | | | | | |  |
